# Blood unconjugated bilirubin and tacrolimus are negative predictors of specific cellular immunity in kidney transplant recipients after SAR-CoV-2 inactivated vaccination

**DOI:** 10.1038/s41598-023-29669-8

**Published:** 2023-05-04

**Authors:** Lei Zhang, Jiaqing Yang, Min Deng, Chuanhui Xu, Changchun Lai, Xuanying Deng, Yan Wang, Qiang Zhou, Yichu Liu, Li Wan, Pingchao Li, Jiali Fang, Jingcai Hou, Xingqiang Lai, Feifei Ma, Ning Li, Guanghui Li, Weiya Kong, Weiting Zhang, Jiali Li, Mibu Cao, Liqiang Feng, Zheng Chen, Ling Chen, Tianxing Ji

**Affiliations:** 1 Kidney Transplant Department, The Second Affiliated Hospital of Guangzhou Medical University, Guangzhou, 510260 People’s Republic of China; 2 Department of Organ Transplantation, The Third Affiliated Hospital of Sun Yat-Sen University, Guangzhou, 510630 People’s Republic of China; 3 State Key Laboratory of Respiratory Disease, Guangzhou Institute of Respiratory Health, The First Affiliated Hospital of Guangzhou Medical University, Guangzhou, 510120 People’s Republic of China; 4 Clinical Laboratory Medicine Department, The Second Affiliated Hospital of Guangzhou Medical University, Guangzhou, 510260 People’s Republic of China; 5 Neurosurgery Department, The Second Affiliated Hospital of Guangzhou Medical University, Guangzhou, 510260 People’s Republic of China; 6 Clinical Laboratory Medicine Department, Maoming People’s Hospital, Maoming, 525000 People’s Republic of China; 7 Department of Pulmonary and Critical Care Medicine, Guangdong Second Provincial General Hospital, Guangzhou, 510317 People’s Republic of China; 8 State Key Laboratory of Respiratory Disease, Guangzhou Institutes of Biomedicine and Health (GIBH), Chinese Academy of Sciences, Guangzhou, 510530 People’s Republic of China; 9 Organ Transplant Department, Zhongshan City People’s Hospital, Zhongshan, 528403 People’s Republic of China; 10 Obstetrical Department, The Second Affiliated Hospital of Guangzhou Medical University, Guangzhou, 510260 People’s Republic of China; 11 State Key Laboratories of Respiratory Diseases, Guangdong-Hong Kong-Macao Joint Laboratory of Infectious Respiratory Disease, Guangzhou Institutes of Biomedicine and Health, Chinese Academy of Sciences, Guangzhou, China; 12 Bioland Laboratory (GRMH-GDL), Guangzhou Institutes of Biomedicine and Health, Chinese Academy of Sciences, Guangzhou, 510530 People’s Republic of China

**Keywords:** Immunology, Risk factors

## Abstract

The immunogenicity of SARS-CoV-2 vaccines is poor in kidney transplant recipients (KTRs). The factors related to poor immunogenicity to vaccination in KTRs are not well defined. Here, observational study demonstrated no severe adverse effects were observed in KTRs and healthy participants (HPs) after first or second dose of SARS-CoV-2 inactivated vaccine. Different from HPs with excellent immunity against SARS-CoV-2, IgG antibodies against S1 subunit of spike protein, receptor-binding domain, and nucleocapsid protein were not effectively induced in a majority of KTRs after the second dose of inactivated vaccine. Specific T cell immunity response was detectable in 40% KTRs after the second dose of inactivated vaccine. KTRs who developed specific T cell immunity were more likely to be female, and have lower levels of total bilirubin, unconjugated bilirubin, and blood tacrolimus concentrations. Multivariate logistic regression analysis found that blood unconjugated bilirubin and tacrolimus concentration were significantly negatively associated with SARS-CoV-2 specific T cell immunity response in KTRs. Altogether, these data suggest compared to humoral immunity, SARS-CoV-2 specific T cell immunity response are more likely to be induced in KTRs after administration of inactivated vaccine. Reduction of unconjugated bilirubin and tacrolimus concentration might benefit specific cellular immunity response in KTRs following vaccination.

## Introduction

Numerous studies have shown that solid organ transplant recipients (SOTRs) requiring lifelong maintenance immunosuppression have a higher risk of SARS-CoV-2 infection and severe COVID-19, even for the less pathogenic omicron variants^1,2^. Given that vaccination is considered as one of the best strategies for curbing the COVID-19 pandemic, several nephrology societies have called for kidney transplant recipients (KTRs) to be prioritized for the administration of SARS-CoV-2 vaccine^3^. The safety and efficacy of various SARS-CoV-2 vaccines in KTRs need to be extensively ascertained since these patients have been excluded from most clinical trials of SARS-CoV-2 vaccines^4^. Clinical trials of mRNA vaccines have demonstrated that humoral and cellular immunity responses were significantly lower in KTRs than in healthy individuals due to their receiving life-long immunosuppression regimens^5,6^. Studies have demonstrated that three doses of mRNA vaccines could further enhance the antibody titers in SOTRs compared with two doses^7^.

Regarding to inactivated vaccine, 11.6 billion vaccine doses have been administered worldwide (45% worldwide), with 65–85% efficacy against symptomatic infection of ancestral strain^8^. Different from other technologies based vaccine, such as protein subunits, viral vectors, and nucleic acid strategies (mRNA and DNA), which are mainly based on the spike protein being key for virus to entering into host cells, the whole virus components are presented to the immune system by administration of inactivated vaccine with an adjuvant^9^. Therefore, multi-protein-specific T cell response could be effectively induced, although the magnitude of spike specific antibody and T cells level was significantly lower that induced by mRNA vaccine in general populations^10^. In addition, inactivated vaccines are relatively cheap and easy to produce, can be stored at 2–8 °C that benefits worldwide vaccine supply, especially in under-developed country. Hopefully, recent data has demonstrated that inactivated vaccines could induce specific cellular immunity response in some KTRs^11^. Moreover, an apparent different proportion of KTRs with positive seroconversion of SARS-CoV-2 specific antibody after second dose of inactivated vaccine have be reported as well^11,12,16,17^. Even that, the risk factors associated with the poor response to COVID-19 vaccination in KTRs are not well-defined^13^.

## Methods

### Subjects

The study was conducted in accordance with the Declaration of Helsinki and was approved by the Ethics Committee of the Second Affiliated Hospital of Guangzhou Medical University (Approval No. 2021-hs-43). The clinical trial protocol was registered with the Chinese Clinical Trial Registry (No. ChiCTR2100049037, Registry’s URL: https://www.chictr.org.cn/listbycreater.aspx). To comparative analysis of the SARS-CoV-2 specific immunity between KTRs and healthy participants (HPs) after administration of inactivated vaccine, KTRs and HPs, who had been administrated with inactivated vaccine or had not been vaccinated, were randomly recruited at the transplant center from June 20, 2021 to August 20, 2021. A total of 163 subjects were enrolled and drawn the whole blood after second dose of inactivated vaccine or before vaccination after obtaining the informed consent. Of the 163 participants, 95 had received two doses of SARS-CoV-2 inactivated vaccine whereas 68 participants were unvaccinated. Of the 95 fully vaccinated participants, 43 were KTRs whereas 52 were HPs. In the unvaccinated group, 38 were KTRs whereas 30 were HPs. None of the participants in the unvaccinated group had a history suggestive of symptomatic COVID-19 infection. In the case of KTRs, the following data was extracted from the records: patient’s clinical data including age, sex, medical history, medication history, kidney transplant time, body mass index, hematologic parameters (white blood cell counts, lymphocyte counts, platelet counts and hemoglobin), hepatic function (alanine aminotransferase, aspartate aminotransferase, and total bilirubin) and kidney function tests (serum creatinine, urine protein, and urine red cells). In the case of vaccinated individuals, the SARS-CoV-2 vaccine brand administered and adverse effects (AEs), if any, were noted.

### Sample processing

Among the vaccinated participants, 10 mL blood was collected from 40 KTRs and 48 HPs between 20 ± 5 days after the second dose of vaccine. Of these, 17 KTRs and 23 HPs also participated in blood collection between 45 ± 10 days after the second dose. Besides, blood was only collected from another 3KTRs and 4HPs between 45 ± 10 days after second dose. In the unvaccinated group, 10 mL blood was drawn for determining the baseline value of SARS-CoV-2 specific humoral and cellular immunity. The plasma was separated by centrifugation (3000 rpm for 15 min) and stored at – 80 °C for anti-SARS-CoV-2 antibody detection. Afterward, an equal amount of Roswell Park Memorial Institute (RPMI) 1640 culture medium (Gibco, USA) was added to the supernatant. Peripheral blood mononuclear cells (PBMCs) were isolated from whole blood samples by Lymphoprep™ density gradient medium (Alere Tech, USA) for T cell immunity response analysis. During the SARS-CoV-2 specific humoral and cellular immunity evaluation, the surveyors were blinded to the source of the sample.

### Anti-SARS-CoV-2 antibody detection

IgG antibodies against receptor-binding domain (RBD), S1 domain of spike protein, (S1) and nucleocapsid proteins (NP) were detected using enzyme-linked immunosorbent assay (ELISA)^14^. Briefly, 100 ng/well of RBD (Dongkang Biotech, China), S1 (Dongkang Biotech, China) or NP (Dongkang Biotech, China) was coated into the ELISA plate well by incubating at 4 °C overnight. After washing with Phosphate Buffered Saline containing 0.05% Tween-20 (PBST) three times, 100 μL of diluted plasma (1:200) was then added into the well of the ELISA plate and incubated at 37 °C for one hour. Following three times washing with PBST, 100 μL of diluted anti-human IgG antibody (1:8000, Southern Biotech, USA) was added into the well of the ELISA plate and incubated at 37 °C for another hour. Then, 50 μL of 3,3′,5,5′-tetramethylbenzidine (TMB) solution (Neobioscience, China) was added after five time washing with PBST, and further incubated at room temperature for 10 min. Finally, 50 μL of 1 M sulfuric acid (H_2_SO_4_) solution was added to terminate the chromogenic reaction. The absorbance at 450 nm was obtained using a microplate absorbance reader (Tecan Sunrise, Switzerland). the vaccinated participants was considered as positivity as the absorbance value higher than mean + 3SD absorbance values of plasma samples from unvaccinated, infection-naïve individuals (including 30 HPs and 38 KTRs).

### Surrogate SARS-CoV-2 neutralization test

The anti-SARS-CoV-2 neutralizing antibody ELISA Kit (Vazyme Biotech, China) was used to qualitatively detect RBD-angiotensin-converting enzyme2 (ACE2) interaction-blocking antibodies. Briefly, 80 μL of horseradish peroxidase (HRP)-conjugated RBD solution was added into a 96-well dilution plate with 8 μL plasma and 72 μL sample dilution buffer, and incubated at 37 °C for 30 min. After that, 100 μL of this plasma/HRP-conjugated RBD mixture was transferred to a microplate coated with ACE2 and incubated at 37 °C for 20 min. After completely washing, 100 μL TMB substrate solution was added with diluted washing buffer, and incubated at room temperature for 15 min. The reaction was stopped with 50 μL of stop solution. Finally, the absorbance at 450 nm was obtained using a microplate absorbance reader (Tecan Sunrise, Switzerland). The inhibition rate was calculated by the following formula: inhibition rate = (1 − absorbance of sample/mean absorbance of negative controls) × 100%. Anti-SARS-CoV-2 neutralizing-antibody positivity was defined by an inhibition rate higher than or equal to 20% according to the manufacturer’s instructions.

### SARS-CoV-2 specific T-cells detection

SARS-CoV-2 spike, or NP-specific T lymphocytes were detected using interferon-γ (IFNγ) enzyme-linked immunospot (ELISPOT) assay. Fresh PBMCs were re-suspended in RPMI 1640 culture medium (Gibco, USA) supplemented with 10% fetal bovine serum (Gibco, USA), 0.55 mM 2-hydroxyethylmercaptan (Gibco, USA), 2 mM l-glutamine (Gibco, USA), 1 mM pyruvate (Gibco, USA), 1% penicillin–streptomycin (Gibco, USA) and 10 mM *N*′-a-hydroxythylpiperazine-*N*′-ethanesulfanic acid (HEPES) (Gibco, USA). The concentration of PBMCs was determined using a hand-held automated cell counter (Millipore, USA). Afterward, 2 × 10^5^ PBMCs were added into each well of an anti-IFNγ pre-coated ELISPOT plate (Dakewe Biotech, China), and co-cultured with overlapping peptide pools of SARS-CoV-2 spike or NP for 24 h. with dimethyl sulfoxide (Sigma, USA) as a negative control (NC). For positive control, 2 × 10^4^ PBMCs stimulated with staphylococcal enterotoxin B (1 µg/mL, Merck, Germany) were adopted. Diluted biotinylated antibody working solution (100 μL) was added to the plate well and incubated at 37 °C for 1 h. Following washing three times, 100 μL streptavidin–horseradish peroxidase working solution was added and incubated at 37 °C for 1 h. After complete washing, 100 μL 3-amino-9-ethylcarbazole (AEC) solution was added and the mixture incubated at room temperature for 30 min. Finally, the spots were counted using the ImmunoSpot^®^ S6 UV Analyzer (Cellular Technology Limited, USA). The spot- forming units (SFU) of each well were determined by subtracting spots of the unstimulated wells from the peptide stimulated wells. The SFU of each sample was calculated using the means of duplicate wells and expressed as SFU/10^6^ PBMC. The threshold for cellular immunity positivity was calculated as the mean + 3 SD SFU/10^6^ PBMC of unvaccinated, infection-naive healthy donors (n = 30) and KTRs (n = 38)^15^. This resulted in cut-off values for Spike, NP, and Spike + NP specific positivity of 69.09, 78.36, and 126.4 SFU/10^6^ PBMC respectively.

### Statistical analysis

Statistical analyses were performed using IBM SPSS Statistics 22.0 or GraphPad prism7.0. The Pearson chi-square was used to test differences in proportions. The t-test or Mann–Whitney U-test was used to explore the difference in continuous variables between two groups. One-way analysis of variance (ANOVA) was applied for comparing the means of continuous variables in the four groups. Paired data was analyzed using paired t-tests. The correlation between anti-SARS-CoV-2 antibody and spike or NP-specific T-cell frequency was determined using the Pearson correlation coefficient. A two-sided p-value ˂ 0.05 was considered statistically significant.

## Results

### Baseline characteristics of the study subjects

The baseline characteristics of participants included in this study are summarized in Table 1. The mean age of KTRs without and with vaccination was 44.57 ± 11.37 and 42.56 ± 9.70 years respectively and was comparable with that of HPs without and with vaccination (44.45 ± 10.63 and 44.00 ± 10.16 years, respectively, *p* = 0.815) (Table 1). A majority of KTRs in the study were male (unvaccinated KTRs 22/30, vaccinated KTRs 35/43). The number of males was significantly higher in KTRs as compared with that in HPs (unvaccinated HPs 17/38, vaccinated HPs 23/58, *p* < 0.0001) (Table 1). The mean time after transplantation in vaccinated KTRs was 68.24 ± 51.41 months, and significantly longer than that in the unvaccinated KTRs (27.37 ± 21.17 months, *p* < 0.0001) (Table 1). The graft type in vaccinated KTRs was comparable to that in unvaccinated KTRs (*p* = 0.483), with most KTRs having had a single kidney transplant (Table 1). The induction agents used in unvaccinated and vaccinated KTRs were comparable (*p* = 0.360), with most KTRs having received anti-thymocyte globulin (ATG), followed by a combination of basiliximab and ATG (Table 1). The majority of vaccinated KTRs had received a uniform immunosuppressive regimen including tacrolimus, mycophenolate mofetil (MMF) and prednisone (40/43), comparable with that in unvaccinated KTRs (29/30, *p* = 0.686) (Table 1). None of the participants had a history suggestive of symptomatic COVID-19. The vaccine brands and AEs in participants are summarized in Table 2. Among the KTRs, 16 (37.2%) of 43 reported at least one AE after receiving the first dose of inactivated vaccine, but this was not significantly different from that in HPs (20/52, 38.5%) (*p* = 0.844) (Table 2). All the reported AEs in KTRs and HPs were mild, transient, and self-limiting.

**Table 1:** Patient’s characteristics.

	HPs without vaccination^#^ (n = 38)	HPs with vaccination* (n = 52)	KTRs without vaccination^#^ (n = 30)	KTRs with vaccination* (n = 43)	p-value
Age (mean year ± SD)	44.45 ± 10.63	44.00 ± 10.16	44.57 ± 11.37	42.56 ± 9.70	0.815
Female/male (%)	21 (55.3)/17 (44.7)	29 (55.8)/23 (44.2)	8 (26.7)/22 (73.3)	8 (18.6)/35 (81.4)	**< 0.0001**
Time since kidney transplant (months)			27.37 ± 21.17	68.24 ± 51.41	**0.002**
Type of graft					
Kidney transplant (%)			27 (90.0)	40 (93.0)	0.483
Simultaneous pancreas-kidney transplant (%)			3 (10.0)	2 (4.7)	
Simultaneous liver-kidney transplant (%)			0 (0.0)	1 (2.3)	
Induction agent used					
ATG (%)			18 (60.0)	30 (69.8)	0.360
Basiliximab + ATG (%)			10 (33.3)	7 (16.3)	
Rituximab + ATG (%)			1 (3.3)	3 (7.0)	
Basiliximab (%)			1 (3.3)	1 (2.3)	
Cyclophosphamide (%)			0 (0.0)	2 (4.7)	
Immunosuppression					
Tacrolimus + MMF + Prednisone			29 (96.7)	40 (93.0)	0.686
Tacrolimus + Mizoribine + Prednisone			1 (3.3)	1 (2.3)	
Tacrolimus + MMF + Rapamycin + Prednisone			0 (0.0)	1 (2.3)	
Cyclosporine A + MMF			0 (0.0)	1 (2.3)	

Significant values are in bold.

*KTRs* kidney transplants recipients, *HPs* healthy participants, *Kidney transplants recipients and healthy participants with administration of inactivated vaccine; ^#^Kidney transplants recipients and healthy participants without vaccination of SARS-CoV-2 inactivated vaccine; *MMF* mycophenolate mofetil, *ATG* anti-thymocyte globulin.

**Table 2:** The adverse effects of kidney transplants recipients and healthy participants after first and second dose of inactivated vaccine.

	HPs (n = 52)	KTRs (n = 43)	p-value
Inactivated vaccine brand of the first dose			
Sinopharm BIBP (%)	21 (40.4)	16 (37.2)	0.752
CoronaVac (%)	31 (59.6)	27 (62.8)	
Inactivated Vaccine brand of the second dose*			
Sinopharm BIBP (%)	5 (9.6)	16 (37.2)	0.001
CoronaVac (%)	47 (90.4)	27 (62.8)	
Time interval between first dose and second dose (day)*	26.69 ± 9.02	26.56 ± 8.15	0.940
Adverse effects after first dose			
No abnormalities (%)	32 (61.5)	27 (62.8)	0.844
Pain at injection site (%)	11 (21.1)	11 (25.6)	
Fatigue (%)	7 (13.5)	4 (9.3)	
Dizzy (%)	1 (1.9)	1 (2.3)	
Allergy (%)	1 (1.9)	0 (0.0)	
Adverse effects after second dose*			
No abnormalities (%)	37 (71.2)	28 (67.4)	0.569
Pain at injection site (%)	10 (19.2)	7 (16.3)	
Fatigue (%)	4 (7.7)	5 (11.6)	
Dizziness (%)	0 (0.0)	1 (2.3)	
Diarrhea (%)	0 (0.0)	1 (2.3)	
Runny nose (%)	1 (1.9)	0 (0.0)	

*KTRs* kidney transplants recipients, *HPs* healthy participants.

### Humoral immunity response of KTR after vaccination

**Figure 1:**
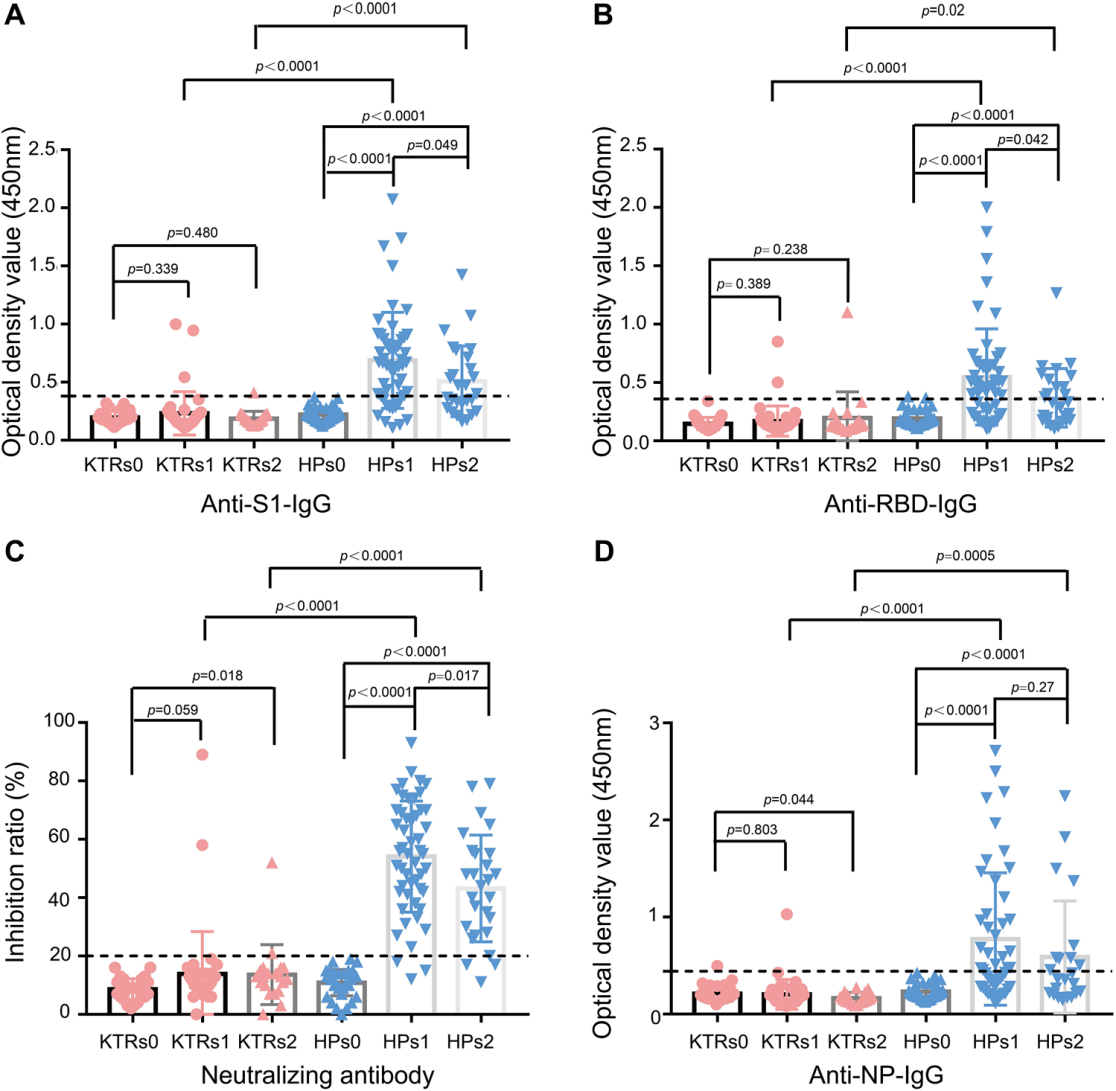
Anti-SARS-CoV-2 antibodies in kidney transplant recipients (KTR) and healthy participants (HPs) at 20 ± 5 days (KTRs1 and HPs1) and 45 ± 10 days (KTRs2 and HPs2) after the second dose of SARS-CoV-2 inactivated vaccine compared with KTRs or HPs without vaccination (KTRs0 and HPs0). (**A**) The optical density of anti-S1 IgG antibody in KTRs and HPs without vaccination and in 20 ± 5 days and 45 ± 10 days after the second dose of SARS-CoV-2 inactivated vaccine. (**B**) The optical density of anti-receptor binding domain (RBD) IgG antibody in KTRs and HPs without vaccination and in 20 ± 5 days and 45 ± 10 days after the second dose of SARS-CoV-2 inactivated vaccine. (**C**) The RBD-Angiotensin I Converting Enzyme 2 (ACE2) interaction blocking antibody in KTRs and HPs without vaccination and in 20 ± 5 days and 45 ± 10 days after the second dose of SARS-CoV-2 inactivated vaccine. (**D**) The optical density of anti-receptor binding domain (RBD) IgG antibody in KTRs and HPs without vaccination and in 20 ± 5 days and 45 ± 10 days after the second dose of SARS-CoV-2 inactivated vaccine. The horizontal dotted line indicates the cut-off value for positivity. The cut-off value was calculated using mean + 3 standard deviation (SD) optical density of plasma samples from HPs and KTRs without vaccination.

Antibody response to SARS-CoV-2 was assessed in individuals 20 ± 5 days and 45 ± 10 days after the second dose of inactivated vaccine. Anti-S1 antibody IgG was effectively induced in most HPs after two doses of inactivated vaccine (Fig. 1A), with 77.1% (37/48) being positive between 20 ± 5 days after the second dose, and 51.9% (14/27) being positive in 45 ± 10 days after the second dose (Table 3). The blood anti-S1 antibody IgG level in KTRs was significantly lower than in HPs (*p *˂ 0.0001) (Fig. 1A), with 7.5% (3/40) of KTRs having anti-S1 antibody IgG positivity in 20 ± 5 days after the second dose, and 5.0% (1/20) in 45 ± 10 days after the second dose (Table 3). Similarly, seroconversion for anti-RBD IgG antibody was observed in most of the HPs but only in two KTRs after the second dose of inactivated vaccine (Fig. 1B and Table 3). RBD-ACE2 interaction-blocking assay was performed to further determine the virus neutralizing antibody level in HPs and KTRs after the second dose of inactivated vaccine. The results suggest that 93.8% (45/48) HPs developed virus-neutralizing antibody after two doses of inactivated vaccine, however, only 5% (2/40) KTRs developed virus-neutralizing antibody after two doses of inactivated vaccine (Fig. 1C, Table 3). Similarly anti-NP-antibody IgG was increased in most of the HPs after two doses of inactivated vaccine (Fig. 1D, Table 3). However, almost all the KTRs had a blunted seroconversion of anti-NP-antibody IgG after two doses of inactivated vaccine (Fig. 1D, Table 3). Statistical analysis was carried out to study the effect of gender and vaccine brand as factors for seroconversion in HPs. The results showed that the anti-RBD-IgG and neutralizing antibody positivity rate (PR) in females was significantly higher than that in males (Supplementary Table 1). Anti-NP IgG PR in HPs receiving the second dose of the vaccine brand Sinopharm BIBP was higher than those who had received CoronaVac as the second dose. (Supplementary Table 2). These data indicate that gender and brand of vaccine have some effect on the immunogenicity. In summary, there was good antibody response in immunocompetent individuals after two doses of inactivated vaccine, but this was not the case with KTRs.

**Table 3:** The spike or nucleocapsid protein specific IgG antibody positive rate in kidney transplants recipients and healthy participants after second dose of inactivated vaccine.

	KTRs	HCs	p-value
20 ± 5 days after 2nd dose	(n = 40)	(n = 48)	
Anti-S1 IgG antibody positive (%)	3 (7.5)	37 (77.1)	**< 0.0001**
Anti-RBD IgG antibody positive (%)	2 (5.0)	32 (66.7)	**< 0.0001**
Neutralizing antibody positive (%)	2 (5.0)	45 (93.8)	**< 0.0001**
Anti-NP IgG antibody positive (%)	1 (2.5)	26 (54.2)	**< 0.0001**
45 ± 10 days after 2nd dose	(n = 20)	(n = 27)	
Anti-S1 IgG antibody positive (%)	1 (5.0)	14 (51.9)	**0.001**
Anti-RBD IgG antibody positive (%)	1 (5.0)	12 (44.4)	**0.003**
Neutralizing antibody positive (%)	2 (10.0)	24 (88.9)	**< 0.0001**
Anti-NP IgG antibody positive (%)	0 (0.0)	11 (40.7)	**0.001**

Significant values are in bold.

*KTRs* kidney transplants recipients, *HPs* healthy participants, *S1* The S1 domain of the spike protein, *RBD* receptor binding domain, *NP* nucleocapsid protein.

### T cells immunity response of KTR after vaccination

**Figure 2:**
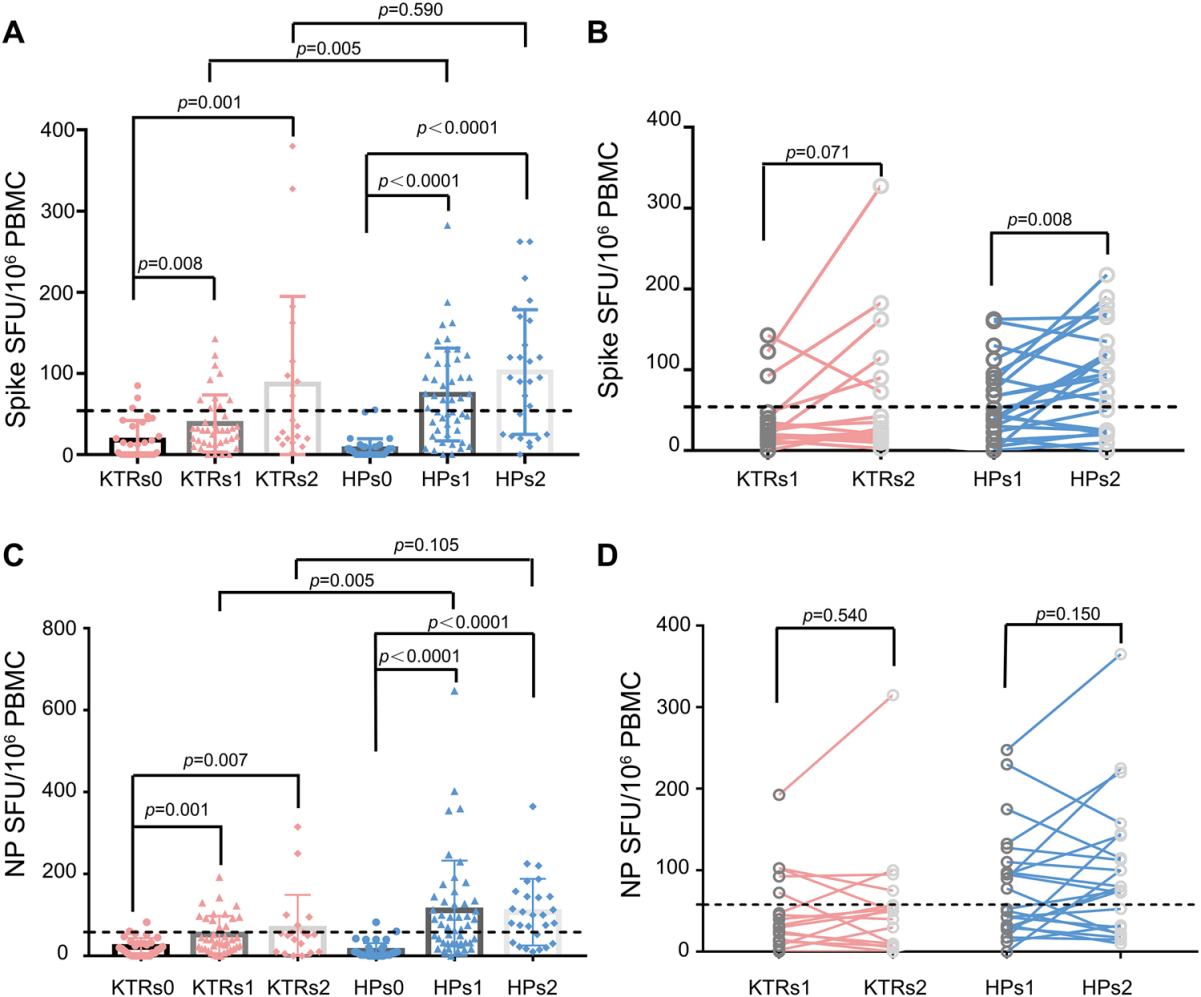
T cell responses to pooled peptides from SARS-CoV-2 spike or nucleocapsid protein (NP) in kidney transplant recipients (KTR) and healthy participants (HPs) at 20 ± 5 days (KTRs1 and HPs1) and 45 ± 10 days (KTRs2 and HPs2) after the second dose of SARS-CoV-2 inactivated vaccine compared with KTRs or HPs without vaccination (KTRs0 and HPs0). (**A**) T cell responses targeted against SARS-CoV-2 spike in KTRs and HPs without vaccination, or at 20 ± 5 days and 45 ± 10 days after the second dose of inactivated vaccine. (**B**) The kinetic of spike specific T cells frequency in paired samples from KTRs and HPs at 20 ± 5 days (KTRs1 and HPs1) and 45 ± 10 days after the second dose of inactivated vaccine. (**C**) T cell responses targeted against SARS-CoV-2 NP in KTRs and HPs without vaccination or at 20 ± 5 days and 45 ± 10 days after the second dose of inactivated vaccine. (**D**) The kinetic of NP-specific T cell frequency in paired samples from KTRs and HPs at 20 ± 5 days (KTRs1 and HPs1) and 45 ± 10 days after the second dose of inactivated vaccine. The dotted line represents the cut-off value, which was calculated using mean + 3 standard deviations (SD) spike or NP-specific T cell frequency of HPs and KTRs without vaccination and COVID-19 history.

The ELISPOT assay was performed to evaluate the cellular immunity against the two major structural proteins of SARS-CoV-2, spike and NP. As shown in Fig. 2, T cells reactive to spike and NP were significantly increased in HPs after the second dose of inactivated vaccine compared with unvaccinated HPs (Fig. 2A,C). Paired analysis demonstrated spike-specific T cell frequency in HPs increased further 45 ± 10 days after the second dose of inactivated vaccine compared with that seen 20 ± 5 days after the second dose, but this was not observed with NP-specific T cells (Fig. 2B,D). An increase in spike or NP-specific T cell frequency was also observed in KTRs after the second dose of vaccine though this response was lower than that observed in HPs (Fig. 2A,C). Paired analysis indicated spike and NP-specific T cell frequency in KTRs 45 ± 10 days after the second dose of inactivated vaccine was not different from that 20 ± 5 days after the second dose (Fig. 2B,D). Both spike and NP-specific T cell frequency in KTRs 20 ± 5 days after the second dose of vaccine were lower than that observed in HPs (Fig. 2A,C). However, both spike and NP-specific T cell frequency in KTRs 45 ± 10 days after the second dose of vaccine were comparable with that observed in HPs (Fig. 2A,C).

**Figure 3:**
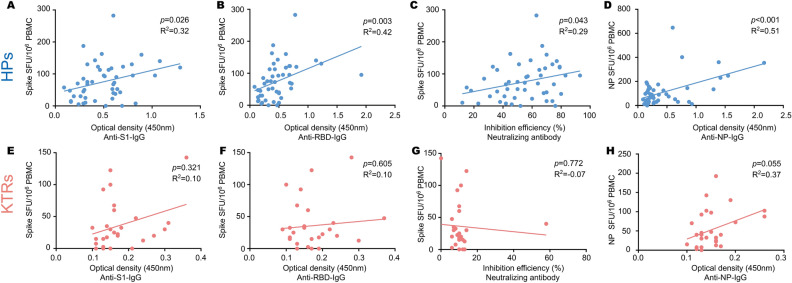
Correlation between T cell immunity response and anti-SARS-CoV-2 antibody level in healthy participants (HPs) and kidney transplant recipients (KTRs) 20 ± 5 days after the second dose of inactivated vaccine. (**A**–**C**) The correlation between spike specific T cells frequency and anti-S1 subunit of the spike protein IgG (**A**), anti-receptor binding domain (RBD) IgG (**B**), neutralizing antibody (**C**) in HPs. (**D**) The correlation between nucleocapsid protein (NP) specific T cells frequency and anti-NP Ig in HPs. (**E**–**G**) The correlation between spike specific T cells frequency and anti-S1 IgG (**E**), anti-RBD IgG (**F**), neutralizing antibody (**G**) in KTRs. (**H**) The correlation between NP specific T cells frequency and anti-NP IgG in KTRs. SFU: Spot forming units.

The positivity rate of spike and NP-specific T cell immunity response in KTRs in 20 ± 5 days after the second dose of vaccine was significantly lower than that observed in HPs (Spike specific T cells: 17.5% versus 47.9%, *p* = 0.006; NP specific T cells: 27.5% versus 47.9%, *p* = 0.050) (Table 4). However, spike-specific T cell immunity response in KTRs in 45 ± 10 days was not statistically different from that in HPs (40.0.0% versus 63.0%, *p* = 0.119) (Table 4). Furthermore, the spike and NP-specific T cell frequency in HPs was significantly related to the anti-RBD/anti-S1/neutralizing antibodies, and anti-NP IgG respectively (Fig. 3A–D). However, this correlation was not found in KTRs (Fig. 3E–H), indicating a dichotomous humoral and cellular immunity response in the KTRs after vaccination. These results indicate that SARS-CoV-2 specific cellular immunity are more likely to be induced in some KTRs after administration of inactivated vaccine.

**Table 4:** The spike or nucleocapsid protein specific T cell positive rate in kidney transplants recipients and healthy participants after second dose of inactivated vaccine.

	KTRs	HPs	p-value
20 ± 5 days after 2nd dose	(n = 40)	(n = 48)	
Spike specific T cell positive (%)	7 (17.5)	23 (47.9)	**0.006**
NP specific T cell positive (%)	11 (27.5)	23 (47.9)	**0.050**
45 ± 10 days after 2nd dose	(n = 20)	(n = 27)	
Spike specific T cell positive (%)	8 (40.0)	17 (63.0)	0.119
NP specific T cell positive (%)	5 (25.0)	16 (59.3)	**0.020**

Significant values are in bold.

*KTRs* kidney transplants recipients, *HPs* healthy participants, *S1* The S1 domain of the spike protein, *RBD* receptor binding domain, *NP* nucleocapsid protein.

### Factors associated with lower SARS-CoV-2 specific T cell immunity response in KTRs

We also explored factors associated with SARS-CoV-2 specific T cell immunity response in KTRs, including age, sex, body mass index, hematologic parameters (white blood cell counts, lymphocyte counts, platelet counts and hemoglobin), hepatic function and kidney function tests, immunosuppressive medications used, induction agent used and comorbidities. The results demonstrated that KTRs with SARS-CoV-2 specific T cells immunity had a higher frequency of females (37.5% versus 7.4% *p* = 0.014), and lower total bilirubin (TB) (9.6 nmol/L versus 12.5 nmol/L, *p* = 0.016), unconjugated bilirubin (UCB) (7.7 nmol/L versus 10.5 nmol/L, *p* = 0.003), blood tacrolimus concentration (BTC) (5.2 ng/mL verves 6.3 ng/mL* p* = 0.001) and longer interval between first vaccination and transplant (67.5 months versus 42 months, *p* = 0.042) compared with KTRs without SARS-CoV-2 specific T cell immunity response after two doses of inactivated vaccine (Table 5). However, there was no association between SARS-CoV-2 specific T cells immunity response in KTRs after two dose of inactivated vaccine with biomarkers including age, body mass index, white blood cell, lymphocyte, and platelet counts, creatinine, alanine aminotransferase, aspartate aminotransferase, conjugated bilirubin, urine protein positivity, urine red cell positivity, immunosuppressive drug administration, induction agent used, transplant graft type, coronary disease, urinary infection and diabetes. Multivariate logistic regression analysis was performed using independent variables with a* p* < 0.1 in the univariate analysis. These included female sex, hemoglobin, TB, UCB, BTC and interval between first dose of SARS-CoV-2 inactivated vaccine and transplant (Table 5). This multivariate logistic regression analysis demonstrated that blood UCB and BTC were significantly negatively associated with SARS-CoV-2 specific T cells immunity response in KTRs (UCB: OR 0.699; 95% CI 0.501–0.976, *p* = 0.036; BTC: OR 0.338; 95% CI 0.116–0.987, *p* = 0.047) (Table 5).

**Table 5:** Comparative analysis of baseline characteristics of kidney transplant recipients with and without SARS-CoV-2 specific T cell immunity response after two doses of inactivated vaccine.

	KTRs without SARS-CoV-2 specific T cells immunity (n = 27)	KTRs with SARS-CoV-2 specific T cells immunity (n = 16)	*p*-value^#^	*p*-value*
Female sex	2 (7.4)	6 (37.5)	0.014	0.156
Age(year)	44.0 (16)	46.0 (20)	0.782	
Body mass index	23.3 (4.5)	21.9 (4.13)	0.152	
White blood cell (× 10^9^)	6.68 (3.17)	7.70 (3.30)	0.821	
Lymphocyte(× 10^9^)	1.54 (1.27)	1.96 (0.83)	0.223	
Platelet (× 10^9^)	196.0 (83)	183 (93)	0.451	
Hemoglobin (g/L)	145 (21)	139 (28)	0.092	0.380
Creatinine (µmol/L)	114.7 (18)	101.8 (39)	0.156	
Alanine aminotransferase (U/L)	22.0 (10)	17.5 (10)	0.209	
Aspartate aminotransferase (U/L)	20.6 (4.60)	19.6 (6.30)	0.980	
Total bilirubin (µmol/L)	12.5 (8.40)	9.6 (4.40)	0.016	0.555
Ungonjugated bilirubin (µmol/L)	10.5 (8.5)	7.7 (3.95)	0.003	0.036
Conjugated bilirubin (µmol/L)	2.4 (2.20)	2.30 (0.9)	0.345	
Urine protein positive	5 (19.2)	1 (5.9)	0.262	
Urine red cells positive	8 (30.8)	5 (29.4)	0.911	
Blood tacrolimus concentration (ng/mL)	6.30 (1.70)	5.20 (1.05)	0.001	0.047
Interval between vaccination and transplant (months)	42.0 (28)	67.0 (75)	0.042	0.816
Immunosuppression				
Tacrolimus + MMF + Prednisone (%)	25 (92.6)	15 (93.8)	0.411	
CsA + MMF + Prednisone (%)	1 (3.7)	0 (0)		
Tacrolimus + Mizoribine + Prednisone (%)	0 (0)	1 (6.3)		
Tacrolimus + MMF + Rapa + Prednisone (%)	1 (3.7)	0 (0)		
Induction agent used				
ATG (%)	17 (63.0)	13 (81.3)	0.226	
Basiliximab + ATG (%)	6 (22.2)	1 (6.3)		
Rituximab + ATG (%)	3 (11.1)	0 (0)		
Basiliximab (%)	0 (0)	1 (6.3)		
Cyclophosphamide (%)	1 (3.7)	1 (6.3)		
Type of graft				
Kidney transplant (%)	25 (92.6)	15 (93.8)	0.238	
Simultaneous pancreas-kidney transplant (%)	2 (7.4)	0 (0)		
Simultaneous liver-kidney transplant (%)	0 (0)	1 (6.3)		
Comorbidity				
Coronary disease (%)	2 (7.4)	0 (0)	0.265	
Urinary infection (%)	1 (3.7)	1 (6.3)	0.702	
Diabetes (%)	4 (14.8)	1 (6.3)	0.397	

Results are expressed as median (interquartile range) and number (%). Continuous data were compared using the Mann–Whitney U-test, and categorical variables with the chi-square. *KTRs* kidney transplant recipients, *MMF* mycophenolate mofetil, *ATG* anti-thymocyte globulin. ^#^*p*-value of Univariate analysis. *p-value of Multiple logistic regression analyses.

## Discussion

Information regarding efficacy and related clinical risk factors of inactivated vaccines in SOTRs is needed to be extensively explored^1,16^. Studies have shown that vaccination of KTRs with mRNA vaccine resulted in lower humoral and cellular immunity response after two doses as compared with healthy individuals^5^. In our study, we found that a positive humoral immunity response was observed in 7.5% KTRs after two doses of inactivated vaccine. This is similar to two previous studies which reported a seroconversion of 7.2% and 9% with two doses of inactivated vaccine^11,12^. However, two other studies have demonstrated that 29% and 58% of KTRs showed seroconversion after two doses of inactivated vaccine, which was comparable to that with two doses of BNT162b2 mRNA vaccine^17,18^. These different results may be related to the different immunosuppressive regime used in the enrolled KTRs in these studies. For example, different from our study with all enrolled KTRs being administrated with tacrolimus and MMF, 14.3% and 4.6% KTRs were not administrated with antimetabolite and calcineurin inhibitors in Seija’s study^18^, which have significant negative impacts on the specific antibody response after vaccination^19,20^.

Nevertheless, the lower SARS-CoV-2 specific immunity response seen in KTRs places them at a higher risk of breakthrough infections with variants of concern including omicron (B.1.1.529)^21,22^. Multiple studies demonstrated heterologous booster with mRNA vaccine or adenovirus vector based vaccine on top of inactivated vaccine could induce higher SARS-CoV-2 specific humoral immunity relative to homologous inactivated vaccine booster in general population^8,23^. In addition, the memory B cells representing long-term immunity could be effectively induced by mRNA vaccine or adenovirus vector based vaccine booster^8,24^. More importantly, recent data demonstrated fourth doses of mRNA vaccine could effectively improve the SARS-CoV-2 ancestral strain and the current prevailing Omicron variants specific humoral and cellular immunity in immunocompromised patients such as elder peoples and chronic lymphocytic leukemia patients^25,26^. Therefore, it is better for these patients to receive a third heterologous booster, and even a fourth dose of SARS-CoV-2 vaccine^7,27–29^. Another potential strategy to improve the immunity response are immunosuppression reduction prior to vaccination^30^. For example, a randomized controlled trial are conducting to explore effects of the interventions (mycophenolic temporary cessation 4 days before (five half-lives) and 1 week (expected antibody response) after vaccination on the SARS-CoV-2 specific humoral and cellular immunity response in KTRs^31^.

Unlike the humoral immunity response, T cell immunity response induced by inactivated vaccine has received scant attention^32^. Consistent with previous studies^33–36^, we too found that HPs and KTRs developed SARS-CoV-2 specific T cell immunity after two doses of inactivated vaccine. Also, the T cell response after two doses of inactivated vaccines in our cohort of KTRs, was higher than the humoral response. This is similar to the findings in studies on mRNA vaccine^37,38^. Different from rapid waning of SARS-CoV-2 specific antibody, SARS-CoV-2 specific T cells increased at 45 ± 10 days after the second dose of inactivated vaccine as compared with those at 20 ± 5 days, stressing the importance of long-lasting cellular immunity in providing a protective role in the face of waning humoral immunity^39^. In addition, the enhanced NP and spike-specific T cell immunity response in KTRs could potentially provide synergistic antiviral effects and prevent severe COVID-19 following SARS-CoV-2 variants of concern infection^20,40,41^. However, the cellular immunity induced by inactivated vaccine is significantly lower than that induced by other vaccines including adenoviral vector vaccine and mRNA^15,42^. Recent data indicated the third homologues booster with inactivated vaccine could not further increase the cellular immunity against SARS-CoV-2^10^. However, third booster with mRNA vaccine or adenovirus vector vaccine could further increase the SARS-CoV-2 specific T cells in healthy individuals vaccinated with two doses of inactivated vaccine^8,24^. Thus, a third dose with heterologous vaccine might be a better strategy for improving T cell immunity response in these patients^7,27^.

Studies, including our own, have demonstrated that there is a discordance between the humoral and cellular immunity response after SARS-CoV-2 vaccination in KTRs^43^. This discordance may be due to the immunosuppressive drugs these patients are on. The triple immunosuppression regime significantly disturbs the interaction between T follicular helper (Tfh) cells and B cells in the germinal center, and suppresses the proliferation of activated T and B cells^44,45^. These processes are pivotal to anti-SARS-CoV-2 specific antibody generation^18,46,47^. In contrast to our study, Bruminhent et al. have shown that the spike and NP specific T cells were enhanced in healthy people after two doses of inactivated vaccine, but not in KTRs^11^. They reported that in KTRs the sum of T cells against spike and NP was 58 SFU/10^6^ PBMCs after two doses of inactivated vaccine^11^. This was lower than that reported in our study (median SFU/10^6^ PBMCs against spike: 67.5, median SFU/10^6^ PBMCs against NP: 76.3), and an another study (median SFU/10^6^ PBMCs against spike: 92, median SFU/10^6^ PBMCs against NP: 7)^33^. Bruminhent et al. also reported that the median numbers of SARS-CoV-2 spike, nucleoprotein, membrane protein, open reading frame (ORF)-3a and ORF-7a proteins (SNMO) peptide pool-specific T cells of 40 SFUs per 10^6^ PBMCs, were significantly lower than previous reports (median 103.9 SFU per 2.5 × 10^5^ PBMC)^35^. These apparent differences between studies of SARS-CoV-2 specific T cells in KTRs may be related to the different immunosuppression protocols used, and technical factors such as cell viability of the isolated PBMCs, the peptide pools used and assay readouts^48^.

In our study we found that specific T cell immunity response to SARS-CoV-2 inactivated vaccine in KTRs was negatively associated with blood unconjugated bilirubin, which is a test for hepatic function^49^. Apart from indicating liver dysfunction, unconjugated bilirubin in physiological ranges can function as an immunosuppressant, by impairment of antigen presentation in macrophages and inhibition of CD^4+^ T cell responses, especially Th1 response (IL-2 and IFN-γ)^50–52^. These mechanisms may explain the lower protective T cell immunity response to SARS-CoV-2 in KTRs following vaccination in those with elevated unconjugated bilirubin. As shown in a previous study^15^, we also found that the T cell immunity response in KTRs after two doses of inactivated vaccine was negatively related to the blood tacrolimus concentration. The underlying mechamism might be related to significant suppressed TCR signaling pathway induced by tacrolimus that further impede the formation of effector and memory T cells against SARS-CoV-2 after vaccination^53,54^. It should be noted that the majority of our patients were treated with mycophenolate mofetil (MMF), which may have contributed to impaired humoral response following vaccination^55^. Whether the low humoral immunity is related to the dysregulation of the T cell response by MMF needs to be further investigated.

Our study has some limitations. Firstly, there was some selection bias towards KTRs, who were performed kidney transplant more than 2 year ago, and had steady physiological parameters after kidney transplant, were more interested in SARS-CoV-2 vaccination. Secondly, our sample size was small and this could have led to an underpowered study. Thus, interpretations of no difference of spike-specific T cell immunity positivity rate 45 ± 10 days after the second dose of vaccine between KTRs and HPs must be made with caution. Thirdly, the pre-vaccination blood sample of KTRs was not collected and hence we were unable to do a paired analysis of the humoral and cellular immunity response before and after vaccination. We tried to overcome this limitation by enrolling 38 HPs and 30 KTRs without vaccination to enable us to determine the threshold of humoral and cellular immunity response. In addition, we included 52 HPs who had received two doses of the inactivated vaccine so that we could compare their humoral and cellular immunity response after vaccination with that of vaccinated KTRs.

In summary, this study demonstrates that SARS-CoV-2 specific cellular immunity response could be effectively induced in some KTRs after administration of two doses of inactivated vaccine. Blood unconjugated bilirubin and tacrolimus levels were negatively associated with SARS-CoV-2 specific cellular immunity response in KTRs. Further prospective studies with an adequate sample size are needed to determine the role of unconjugated bilirubin levels in predicting the cellular response to inactivated vaccines in KTRs.

## Supplementary Information

Supplementary Tables.

## Data Availability

All data generated or analysed during this study are included in this published article.
