# Supplementary material for: Blood unconjugated bilirubin and tacrolimus are negative predictors of specific cellular immunity in kidney transplant recipients after SAR-CoV-2 inactivated vaccination
Source: Sci Rep. 2023 May 4;13:7263. doi: 10.1038/s41598-023-29669-8 (PMC10158706; doi:10.1038/s41598-023-29669-8)
Supplement: Supplementary file 1 — Supplementary Tables. [file 41598_2023_29669_MOESM1_ESM.docx]

Supplemental table1. Comparison of humoral and cellular immunity response between female and male in healthy participants 20 ± 5 days after second dose of inactivated vaccine.

|  | **Female** | **Male** | **p-value** |
| --- | --- | --- | --- |
| **20±5 days after 2^nd^ dose** | **(n=27)** | **(n=21)** |  |
| Anti-S1 IgG antibody positive/negative (%) | 23(85.2) | 14(66.7) | 0.130 |
| Anti-RBD IgG antibody positive/negative (%) | 21(77.8) | 11(52.4) | 0.064 |
| RBD-ACE2 blocking antibody positive (%) | 27(100) | 18(85.7) | **0.043** |
| Anti-NP IgG antibody positive/negative (%) | 14(51.9) | 12(57.1) | 0.715 |
| Spike specific T cell positive/negative (%) | 18(66.7) | 8(38.1) | **0.049** |
| NP specific T cell positive/negative (%) | 18(66.7) | 10(47.6) | 0.184 |
| S1: The S1 domain of the spike protein. RBD:receptor binding domain, ACE2: angiotensin-converting enzyme 2, NP: nucleocapsid protein | | | |

Supplemental table2. Comparison of humoral and cellular immunity response of second dose with Coronavac and Sinopharm BIBP in healthy participants 20 ± 5 days after first or second dose of inactivated vaccine.

|  | **Coronavac** | **Sinopharm BIBP** | ***p*-value** |
| --- | --- | --- | --- |
| **20±5days after 2nd dose** | **(n=43)** | **(n=5)** |  |
| Anti-S1 IgG antibody positive (%) | 33(76.7) | 4(80.0) | 0.870 |
| Anti-RBD IgG antibody positive (%) | 29(67.44) | 3(60.0) | 0.738 |
| RBD-ACE2 blocking antibody positive (%) | 41(95.3) | 4(80.0) | 0.180 |
| Anti-NP IgG antibody positive (%) | 21(48.8) | 5(100.0) | **0.030** |
| Spike specific T cell positive (%) | 22(51.1) | 4(80.0) | 0.221 |
| NP specific T cell positive (%) | 24(55.8) | 4 (80.0) | 0.299 |
| S1: The S1 domain of the spike protein. RBD:receptor binding domain, ACE2: angiotensin-converting enzyme 2, NP: nucleocapsid protein | | | |
